# Origin, Evolution, Breeding, and Omics of Chayote, an Important Cucurbitaceae Vegetable Crop

**DOI:** 10.3389/fpls.2021.739091

**Published:** 2021-09-24

**Authors:** Yu-Ting Pu, Qing Luo, Lin-Hong Wen, Yu-Rong Li, Ping-Hong Meng, Xiao-Jing Wang, Guo-Fei Tan

**Affiliations:** ^1^Key Laboratory of Plant Resource Conservation and Germplasm Innovation in Mountainous Region (Ministry of Education), Guizhou University, Guiyang, China; ^2^Institute of Horticulture, Guizhou Academy of Agricultural Sciences, Guiyang, China

**Keywords:** chayote, genetic breeding, genomic research, chemical composition, pests and diseases

## Abstract

Chayote (*Sechium edule*), a member of the Cucurbitaceae family, is cultivated throughout tropical and subtropical regions of the world and utilized in pharmaceutical, cosmetic and food industries because it is an excellent source of minerals, dietary fibers, protein, vitamins, carotenoids, polysaccharides, phenolic and flavonoid compounds, and other nutrients. Chayote extracts process various medicinal properties, such as anti-cardiovascular, antidiabetic, antiobesity, antiulcer, and anticancer properties. With the rapid advancements of molecular biology and sequencing technology, studies on chayote have been carried out. Research advances, including molecular makers, breeding, genomic research, chemical composition, and pests and diseases, regarding chayote are reviewed in this paper. Future exploration and application trends are briefly described. This review provides a reference for basic and applied research on chayote, an important Cucurbitaceae vegetable crop.

## Introduction

**Figure 1:**
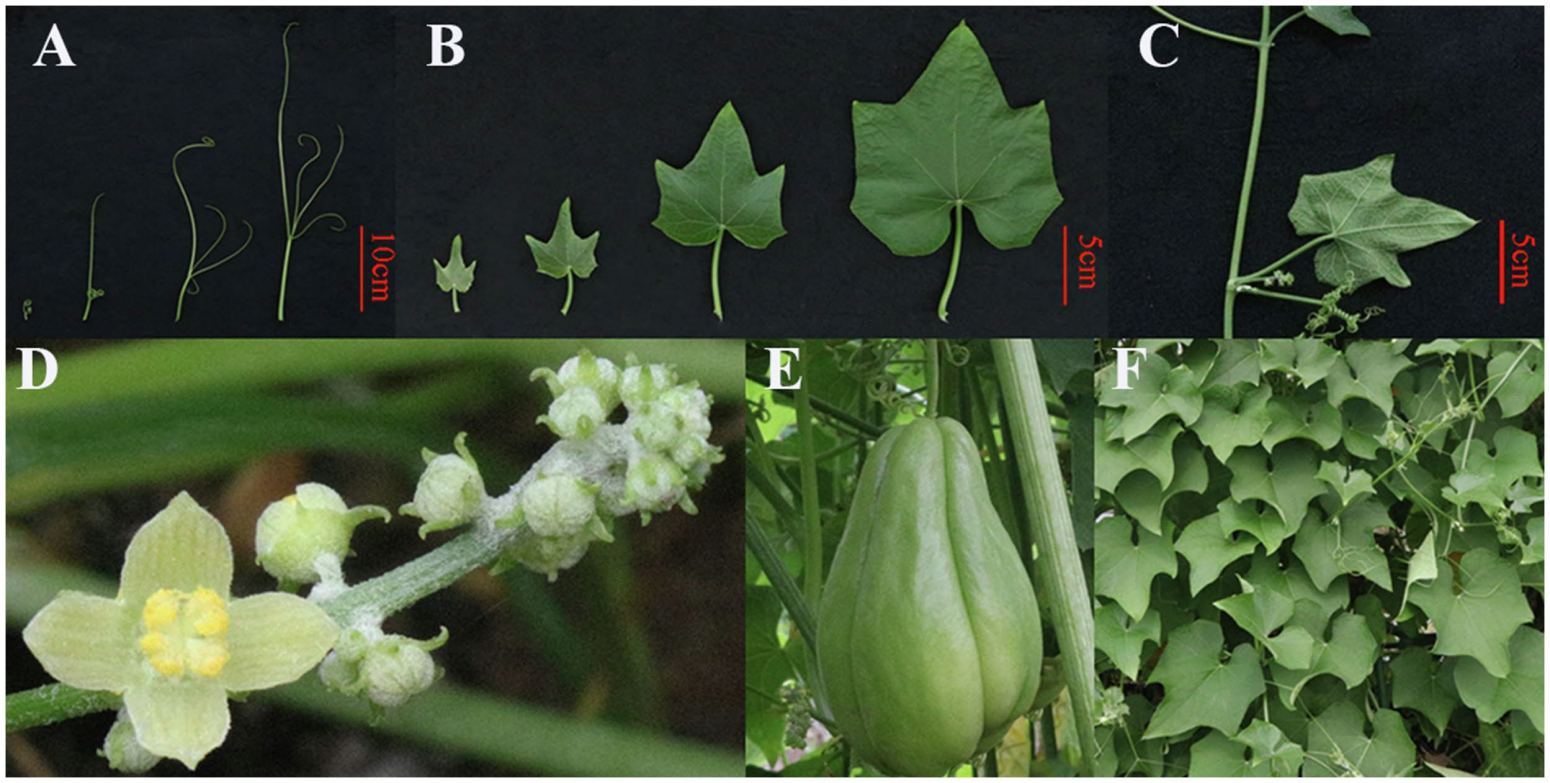
The whiskers **(A)**, leaves **(B)**, stem **(C)**, flowers **(D)**, fruit **(E),** and plants **(F)** of the *Sechium edule*.

*Sechium edule* (Jacq.) Swartz (chayote; Figure 1), an herbaceous perennial climbing plant that belongs to the Cucurbitaceae family (Vieira et al., 2019; Ke et al., 2020), is cultivated in tropical and subtropical areas around the world (Bisognin, 2002; Riviello-Flores Luz et al., 2018). The *S. edule* plant has a tuberous rootstock and heart-shaped leaves (Shiga et al., 2015). *S. edule* is a monoecious plant with male flowers borne in clusters, and females occur singly (Abdelnour and Rocha, 2008; Chakravarty et al., 2019). Chayote fruit, as an important edible organ, has many nutrients needed by the body (Newstrom, 1991; Ruiz-López et al., 2010; Castro Rodríguez et al., 2015; Díaz-de-Cerio et al., 2019; Taynath et al., 2020; Sudargo et al., 2021). Moreover, other parts of *S. edule* plant, such as stems, tender leaves, and tuberous roots, are typically considered to be an important component of human diets (Jena et al., 2018; Vieira et al., 2019).

Chayote is rich in vitamins, proteins, polysaccharides, phenolic compounds, cucurbitacins. Chayote can also utilized in food, pharmaceutical, and cosmetic industries due to numerous health-promoting ingredients (Barba et al., 2014; Loizzo et al., 2016; Coronel et al., 2017; Rosado-Pérez et al., 2019). With the rapid advancements of molecular biology and sequencing technology, including genome sequencing, transcriptome sequencing, proteome sequencing, small RNA sequencing, and digital expression profile, large amount of data resource and research methods have been applied to study chayote plant. More and more attention has been attracted in exploring the molecular mechanisms of these health-promoting ingredients. However, so far, there is no available updated review that focuses on all aspects of this valuable vegetable. This prompted us to review the updated information of *S. edule* plant, including its origin and distribution, chemical composition, pests and diseases, breeding, omics research, and medicinal value, reported by previous studies.

## Biology and Origin

### Biology

The chayote plant (*S. edule*) is a perennial and monoecious climber with thickened roots, slender stems, and tendrils (Saade, 1996; Lim, 2012). The leaves of *S. edule*, located on sulcate petioles, are ovate-cordate to suborbicular and contain three to five separate tendrils, and the edges of leaves have minutely denticulate margins and contain three to five separate tendrils (Singh, 2007; Lim, 2012; Vieira et al., 2019). The flowers of *S. edule* are unisexual and contain both male and female reproductive units on the same plant (Quezada-Euán, 2018; Newstrom, 2019b). The clustered male flowers contain five stamens, which are distributed at intervals along the rachis. The female flowers are normally on the same axilla as the staminate flowers typically found singly (Chakravarty, 1990; Joshi et al., 2020). *S. edule* fruit developed from the ovary and grow singly on the plants. Fruit shape and size, as well as spine number and type on the fruit, vary in different varieties (Saade, 1996). Moreover, the skin of the smooth and prickly chayote also varies in color from yellowish-white to pale green to dark green (Cook, 1901; Newstrom, 1991; Moreira, 2015; Newstrom, 2019a). The seed is compressed and ovoid and contains soft and smooth testa (Singh, 2007).

### Origin

The time frame and geographical location(s) of the *S. edule* plants remain unclear. Unlike other crops, there is no archaeological evidence to indicate how long *S. edule* has been cultivated. Previous studies revealed that the chayote originated from Mexico and Guatemala based on its concentrations of genetic diversity (Newstrom, 1991). However, Moreira, 2015 confirmed that Guatemala was not the place of origin of chayote based on the pan-etymological analysis. It is more likely that chayote was first cultivated or identified in the twelfth century BCE by Otomanguean speakers in southern Mexico (Moreira, 2015). Moreover, archaeological evidence supports the theory that the Mayans cultivated chayote as early as the eighth century. The closest morphological variants to chayote, known as the “wild” types of *S. edule* and *Sechium chinantlense*, are found in southern Mexico (Moreira, 2015). The chayote was introduced into the United States in the eighteenth century before its introduction to Europe and then Africa (Singh, 2007). During the same century, the chayote was introduced into southeast Asian countries (Saade, 1996). It was not introduced to China until the nineteenth century and is primarily distributed in all the provinces of southern China (Walters, 1989).

## Nutritional Properties

All parts of *S. edule* plant can be consumed, including fruits, young leaves, shoots, and tuberous roots, and they primarily used for culinary purposes owing to their rich nutrients (Shiga et al., 2015; Sakung et al., 2020). A summary of the nutritional composition of the different edible parts of chayote is presented in Table 1.

**Table 1:** Main nutrients of *Sechium edule*.

Secondary metabolites	Roots	Stems	Leave	Fruits	Seeds
Carbohydrates (g)	17.80	4.70	–	4.51	60.0
Soluble sugars (g)	–	0.30	–	1.66	4.20
Starch (g)	0.70	13.60	–	0.20	1.90
Fiber (g)	0.40	1.20	–	1.70	–
Lipids (g)	0.20	0.40	2.32	0.13	–
Nonpolar lipids (g/100g Lipid)	–	–	40.2	–	–
Glycerolipids (g/100g Lipid)	–	–	30.8	–	–
Phospholipids (g/100g Lipid)	–	–	29.0	–	–
Proteins (g)	2.00	4.00	2.69	0.82	5.50
Amino acids (mg/g Protein)				Fruit	Seeds
Lysine	–	–	5.93	0.42	1.53
Histidine	–	–	2.20	0.23	0.67
Arginine	–	–	4.10	0.54	2.50
Aspartic acid	–	–	1.24	1.45	2.11
Threonine	–	–	2.78	0.64	0.88
Serine	–	–	1.03	0.73	1.55
Glutamic acid	–	–	12.15	1.97	5.25
Proline	–	–	6.00	0.69	0.97
Glycine	–	–	11.01	0.65	0.96
Alanine	–	–	11.36	0.80	1.57
Cysteine	–	–	-	0.04	0.10
Valine	–	–	8.34	0.99	1.74
Methionine	–	–	5.25	–	0.27
Isoleucine	–	–	0.60	0.70	1.30
Leucine	–	–	9.89	1.21	2.69
Tyrosine	–	–	2.61	0.50	0.76
Phenylalanine	–	–	5.10	0.75	1.81
Vitamins				Fruit	
Thiamine (mg)	0.05	0.08	–	0.03	–
Riboflavin (mg)	0.03	0.18	–	0.03	–
Niacin (mg)	0.90	1.10	–	0.47	–
Pantothenic acid (mg)	–	–	–	0.25	–
Pyridoxine (mg)	–	–	–	0.08	–
Folic acid (μg)	–	–	–	93	–
Vitamin C (mg)	19	16	–	7.70	–
Vitamin A (UI)	–	615	–	56	–
Vitamin E (mg)	–	90	–	0.12	–
Vitamin K (μg)	–	–	–	4.10	–

### Roots of *S. edule*

The tuberized roots of *S. edule*, known as ichintal, are widely consumed in Latin America (Aung et al., 1990; Melo et al., 2006). These roots are generated after the first year of growth and contained a substantial amount of quality starch and fiber. Shiga et al. (2015) have shown that the concentration of starch in the tuberized roots of *S. edule* can be as high as 65% (w/w; dry weight, DW). The cell wall structure of tuberized roots of *S. edule* is similar to those of potato, sweet potato, and cassava, which are mostly composed of high amounts of cellulose (92% the fresh sample and 88% in cooked samples), hemicellulose, medium amounts of pectin, and a high amount of glucose (47%). Therefore, the tuberized roots of *S. edule* are commonly used as an excellent substitute for the starch produced from potatoes and corn. In addition, the fresh tuberized roots of *S. edule* are an excellent resource of ascorbic acid (19mg/100g, DW) and phosphorus (34mg/100g, DW; Shiga et al., 2015; Table 1).

### Seeds of *S. edule*

The seeds of *S. edule* are composed of endocarp, endosperm, and cotyledons. The moisture content in mature seeds of *S. edule* differs from those of other crop seeds (Luna-Solano, 2018). The seeds of *S. edule* are rich in nutrients, such as proteins (5.5g/100g, DW), carbohydrates (60g/100g, DW), lipids (0.4g/100g, DW), and eight essential amino acids including leucine, arginine, phenylalanine, valine, lysine, isoleucine, threonine, and histidine (Vieira et al., 2019).

### Leaves and Stems of *S. edule*

The tender leaves and stems of cultivated *S. edule* are frequently consumed daily (Booth et al., 1992; Ordonez et al., 2006). The tender leaves of *S. edule* contain a considerable amount of protein (2.69~4.88g/100g DW), pectin (0.45g/100g DW), lipids (0.4~2.32g/100g, DW; Vieira et al., 2019). The main fatty acids include the linolenic (42.1–76.7%), palmitic (13.7–38.5%), and linoleic (5.7–15.3%) acids, which are richly distributed in the tender leaves (Wijaya, 2013; Del Ángel-Coronel et al., 2018). The content of 13 amino acids (lysine, histidine, arginine, threonine, glutamic acid, proline, glycine, alanine, valine, methionine, leucine, tyrosine, and phenylalanine) in tender leaves was significantly higher than those in fruits and seeds (Wijaya, 2013). The stems of *S. edule* plant also contain dietary fiber (1.20~21.70%), protein (4%), niacin (1.10mg/100g, DW), vitamin A (615 UI/100g, DW), and vitamins E (90mg/100g, DW). Thus, the tender vine is also widely consumed all around the world owing to its tenderness, flavor, short growth cycle, and rich nutrients (Chadha, 2009; Rohini et al., 2017).

### Fruit and Peels of *S. edule*

Chayote fruit is an important part of the human diet (Dire et al., 2010). The contents of calories and lipids in chayote fruit (19~31kcal/100g, DW and 0.10~0.30g/100g, DW, respectively) are relatively lower than those in the young stems (60kcal/100g, DW and 0.4g/100g, DW, respectively) and tuberous root (80kcal/100g, DW and 0.20~0.33g/100g, DW, respectively). Chayote fruits are rich in complex carbohydrates, including dietary fiber (0.40~7.60g/100g, DW) and starchy (0.2~1.56g/100g, DW; Shiga et al., 2015). Moreover, the fiber content of chayote fruit is higher than that in tuberous roots, but lower than those in the stems and leaves, and the starch contents in chayote fruit are relatively lower than those in other organs (Shiga et al., 2015). Chayote fruits are rich in vitamin C (7.7~20mg/100g, DW), vitamin E (0.12mg/100g, DW), and folate (93 ug/100g, DW; Fauziningtyas and Ristanto, 2020). In addition, the chayote fruit contains several minerals, including potassium (125~338mg/100g, DW), calcium (12~25mg/100g, DW), phosphorus (4~60mg/100g, DW), and magnesium (12~15.4mg/100g, DW).

## Bioactive Compounds and Functional Food Potential

### Sterols and Cucurbitacins

Akihisa et al. (1986) determined the total sterol content in aerial sections (16mg/100g, DW) and the pericarp (38mg/100g, DW) of *S. edule* plants purchased locally in Japan. Gas liquid chromatography technology was used to separate and identify 23 sterols and 14 triterpene alcohols from the sterol mixture (Akihisa et al., 1986). Cucurbitacins, as a group of tetracyclic triterpenoids, serve as heterologous chemical pheromones that protect the plants from external biological stress (Chen et al., 2012; Odeh et al., 2014; Chen, 2015). Previous studies demonstrated that numerous cucurbitacins obtained from natural sources exhibited potent cytotoxic activity and dramatically inhibited the growth and proliferation of cancer cells (Lan et al., 2013; Samuel, 2019; Shamran and Abed, 2020). To date, over 40 new cucurbitacins and their derivatives have been identified from the Cucurbitaceae crops and other plants (Riviello-Flores Luz et al., 2018). Cadena Iñiguez et al. (2011) identified several cucurbitacins from eight varieties of chayote, including Cu, B, Cu, E, Cu, P, and Cu, glycosides, dihydrocucurbitacin, dihydroisocucurbitacin-I, glycocucur bitacin-I, dihydrocucurbitacin-D, isocucurbitacin-D, dihydroiso cucurbitacin-E, hydrocucurbitacin-E, isocucurbitacin-B, dihy droisocucurbitacin-B, cucurbitacin-L, cucurbitacin-E, and cucurbitacin-B. Previous studies revealed that the content of cucurbitacin in different varieties of chayote varied. The content of cucurbitacin in wild-type fruit (1.456mg/g fresh weight, FW) was higher than that in the commercial variety “Virens levis” (0.116mg/g, FW). Moreover, the yellow varieties contained low concentrations of cucurbitacins in comparison with the domesticated green varieties (Cadena Iñiguez et al., 2011).

### Polyphenol Compounds

Polyphenolic compounds not only protect plants from diseases and insect attack, but also possess antiallergic, anti-inflammatory, antiviral, anticarcinogenic, and hypoglycemic properties (Kratchanova et al., 2010; Chun-Lin et al., 2012; Lattanzio, 2013). Siciliano et al. (2004) had reported that the content of anthraquinones, phenolic acids, coumarins, and flavonoids like anthocyanins was in the leaves (0.35g/100g, DW), followed by the roots (0.31g/100g, DW) and stems (0.19g/100g, DW). The content of apigenin 6-C-ß-D-glucopyranosyl-8-C-ß-D-apiofuranoside and diosmetin 7-O-rutinoside (0.133g/100g DW and 0.012g/100g DW) was higher than those in the leaves and roots. The roots of *S. edule* contained the highest amount of vicenin-2 (0.147g/100g, DW) and vitexin (0.151g/100g, DW), and the leaves possess the highest amount of luteolin 7-*O*-rutinoside (0.141g/100g, DW), luteolin 7-*O*-β-D-glucoside (0.135g/100g, DW) and apigenin 7-*O*-rutinoside (0.018g/100g, DW). Ragasa et al. (2014) found that trans-cinnamic acid, phenylacetic acid, 3-octadecenoic acid, trilinolenin, and α-linolenic acid are present in chayote leaves. Chao et al. (2014) revealed that the phenolic content in leaves of green chayote (0.262g GAE/100g, DW; GAE: gallic acid equivalents) was higher than that in the leaves of yellow chayote (0.063g GAE/100g, DW). Moreover, Siciliano et al. (2004) observed that the relatively high content of flavonoids is widely distributed in the tuberized roots of *S. edule* (0.31g/100g, DW).

### Vitamins, Carotenoids, and Polysaccharides

Chayote fruit is a much more impressive source of vitamins (Kumar et al., 2017; Fadliya et al., 2018; Daulay et al., 2021). Folate, also called vitamin B9, reduces the risk of birth defects of the brain and decreases the risk of preeclampsia and early labor (Fauziah et al., 2019; Purba et al., 2019; Fauziningtyas and Ristanto, 2020). The folate only exist in the fruits with high content, which was further determined before and after the cooking process (93ug/100g, DW and 18ug/100g, DW, respectively; Coronel et al., 2017). Vitamin A and vitamin E are also primarily distributed in chayote fruit, and they play important roles in maintaining good vision, reproductive health, healthy blood, and normal brain and skin (Vieira et al., 2019). A study reported that the stems of *S. edule* plants contain the highest content of vitamin A (615 Ul/100g, DW) and vitamin E (90mg/100g, DW), followed by the fruit (vitamin A, 50 Ul/100g DW and vitamin E, 4.7mg/100g, DW). Vitamin C is widely distributed in the fruit, peel, and leaves of *S. edule*, with the highest content in the peel (51.6mg/100g, DW), followed by the fruit (5.5mg/100g, DW) and leaves (4.6mg/100g, DW; Coronel et al., 2017).

Carotenoids are the most widely distributed pigments and naturally exhibit red, orange, and yellow colors. The type and content of carotenoids have been measured in different organs of the *S. edule* plant. Medina (2019) reported that the content of total carotenoids and β-carotene in the fruit peel was 1.7mg/100g, DW and 0.36mg/100g, DW, respectively. The content of lutein and ß-carotene in the leaves (7.4mg/100g, FW and 4.4mg/100g, FW) was determined by Sriwichai et al. (2016).

Polysaccharides, as a long chain of carbohydrates, are used to provide structural support, store energy, and communicate signals, which include starch and non-starch polysaccharides (cellulose, gums, and hemicelluloses). The content of starch in chayote fruit is 0.2~1.56g/100g, DW, with the highest amount in chayote root (13.6~72.8g/100g, DW), followed by seeds (1.9g/100g, DW) and stems (0.7g/100g DW). The content of non-starch polysaccharides in chayote fruit is 0.4~7.6g/100g, DW, with the highest amount in fruit peel (45.2g/100g, DW), followed by the stem (21.7g/100g, DW) and roots (16g/100g, DW; Shiga et al., 2015).

Non-starch polysaccharides are comprised of water-soluble polysaccharides and water-insoluble polysaccharide. Shiga et al. (2015) indicated that the water-soluble polysaccharides in chayote fruit are composed of arabinose (31~36mol%. 1mol%=10,000ppm), galactose (31~35mol%), glucose (11~15 mol%), galacturonic acid (9~11mol%), and mannose (5~7mol%). In contrast, the water-insoluble polysaccharides in chayote fruits are mainly composed of galactose (60mol%), and lesser amounts of arabinose (11mol%), xylose (7mol%), and glucuronic acid (7mol%).

### Pharmacological Properties

Bioactive compounds can be divided into two groups: nutrients and nutraceuticals. Nutraceuticals, derived from “nutrition” and “pharmaceuticals,” are non-nutritive plant chemicals with high antioxidant activity that sustain or promote health and have attracted the attention of the food and pharmaceutical industries (Meisel, 1997; Nasri et al., 2014; Santini and Novellino, 2017; Ghani et al., 2019). The effects of chayote on anti-cardiovascular diseases are primarily owing to its flavonoids (Juan et al., 2001; Lamuela-Raventos, 2005; García-Lafuente et al., 2009), which prevent atherosclerosis and fatty liver by reducing the contents of serum lipids and cholesterol (Meisel, 1997). Hydroalcoholic extracts of the root, pulp, and peel display antihypertensive activity owing to the presence of cinnamic acid methyl ester, coumaric acid, and vitexin (Vieira et al., 2019). The polysaccharides and phenolic composition, particularly flavonoids, play important roles in hypoglycemic effects (Elavarasan et al., 2016). The oral administration of chayote fruit juice was reported to promote the normalization of oral glucose tolerance ability and reduce oxidative stress (Mukherjee et al., 2013; Tiwari et al., 2013; Loizzo et al., 2016; Sangma et al., 2019).

Extracts of the shoots, including caffeic acid and hesperetin, increase the activity of amp-activated protein kinase and decrease the activities of lipogenic-related enzymes involved in the regulation of metabolism of hepatic lipids (Wu et al., 2014). Ordoñez et al. (2009) revealed that the increasing interest in the health benefits of flavonoids in *S. edule* was owing to its potent antioxidant and free radical scavenging abilities. Moreover, the bioactive polysaccharides in the *S. edule* plant, mostly represented by arabinans and homogalacturonans, have a high amount of antioxidant activity, which can modulate the functions of macrophages (Ordonez et al., 2006; Castro-Alves and Do Nascimento, 2016). Arabinogalactans, arabinoxylans, and glucuronoxylans in chayote pulp had a potent immunomodulatory effect (Classen et al., 2006; Duan et al., 2010), which was associated with their side-chains. For instance, type II arabinogalactan has an immunomodulatory effect associated with their branched structure. In addition, further analysis showed that crude chayote pectin has a cell protective effect (Shiga et al., 2015). The antiulcer activity of ethanolic extract of chayote fruit was owing to a reduction in total acidity and free acidity and an increase in the pH of gastric secretion (Sateesh et al., 2012). Six saponins isolated from the fruits and aerial parts of the *S. edule* plant demonstrated antiproliferative activity against MK-1, HeLa, and B16F10 tumor cells. Sechiumin protein, isolated from seed aqueous extracts, possesses the ability to inactivate ribosomes and has the potential of being chemotherapeutic (Ordoñez et al., 2009; Lim, 2012; Tamayo et al., 2016). Moreover, chayote fruit extracts have a positive impact on the treatment of acute myeloid leukemia, which also reduces the viability of cells and induces apoptosis (Aguiñiga-Sánchez et al., 2015). In addition, extracts of the chayote fruits were reported to inhibit the growth of pathogens, such as *Escherichia coli*, *Klebsiella pneumoniae*, *Pseudomonas mirabilis*, and *Enterobacter cloacae* (Ordoñez et al., 2003; Zampini et al., 2009). Although the pharmacological properties of *S. edule* plant were widely investigated and verified through *in vitro* and *in vivo* experiments, the molecular mechanism still remains unclear.

### Uses of *S. edule*

All parts of the *S. edule* plants are useful. The fruits, young shoots, and tuberous roots are used as vegetables, and the leaves and fruits are also used as medicine. Moreover, the tuberous roots of *S. edule* can be used as a good substitute for potato, cassava, and wheat in diverse products (Aila-Suárez et al., 2013), Moreover, the tuberous roots of *S. edule* can be used as a good substitute for potato, cassava, and wheat in diverse products (Shiga et al., 2015), sweets, and pickles (Nuwamanya et al., 2010). *S. edule* plants were also used as a traditional medicine to treat several diseases due to the presence of secondary metabolites (Aung et al., 1990; Lombardo-Earl et al., 2014). The fruits of *S. edule* are used for weight loss owing to their low calorie count and high amount of fiber, and the leaves are used to dissolve kidney stones (Jensen and Lai, 1986; Luna-Solano, 2018). Moreover, the *S. edule* plants are extensively utilized in the cosmetic and toiletry industries, including the production of skincare products, cosmetics, and pharmaceutical products (Wang et al., 2017). More recently, Metral et al. (2018) observed that chayote fruit extract protected keratinocytes against UVA-induced cytotoxicity and decreased the intracellular amounts of reactive oxygen species. In addition, Elavarasan et al. (2016) synthesized zinc oxide nanoparticles from the leaf extract that were significantly cytotoxic to MCF-7 breast cancer cells.

## Breeding, Biotechnology and Breeding

### Breeding

Physical characteristics of the chayote fruit include its shape, size, surface texture, fruit color, and yields (Newstrom, 2019a). All of this would seem to point to the fact that this genus appears to have an enormous potential for genetic resources. However, the lack of knowledge in this field is primarily owing to the need for experimental materials. Efforts to conserve and study these resources have had less than satisfactory results. The endocarpic and precocious germination of the *S. edule* seed seriously hinders the breeding process. Commercial chayote varieties suffer from pests and diseases and a need to maintain fruit quality. Therefore, it is necessary to develop a breeding plan that takes these two aspects into consideration. So far, only written proposals associated with these two aspects have been issued. Newstrom (1986) recommended the development of two different breeding lines: unflavored fruits for industrial purposes and tasty fruits to use as vegetables. Aung et al. (1990) proposed to screen chayote varieties based on root characteristics, such as root length and starch content, associated with chayote types and their growth conditions, which were further used to evaluate wild species against some of the targets described above, particularly those related to disease resistance.

### Molecular Genetic Markers

Molecular genetic markers are used to identify various genetic variations that link genetic traits to potential genomic variations (Domoney et al., 2006; Duran et al., 2009; Perez-de-Castro et al., 2012). The development of different genetic markers and advancement in sequencing technologies has promoted the improvement of crops (Wani et al., 2007; Bhat et al., 2010; Hawkesford, 2012). Genetic markers are classified into classical markers, including morphological, cytological and biochemical markers, and DNA markers, such as RFLP, AFLP, SSRs, and SNP (Lewis et al., 2008; Wang et al., 2013). Genetic markers have also been applied in basic research on the utilization, and breeding of *S. edule*. In an effort to study the plant’s genetic diversity and germplasm identification, 42 accessions of *S. edule* from Costa Rica were successfully characterized using isozyme markers (Vitomir et al., 2012). Joshi et al. (2020) classified 12 accessions of *S. edule* from on-farm and on-station into three categories using nine morphological and 20 RAPD markers. The genetic variation of 36 accessions of *S. edule* collected across 12 states in India was analyzed using directed amplification of minisatellite DNA (DAMD) technology and morphological traits (Jain et al., 2017). In addition, the genetic variations of 74 landraces of *S. edule* collected from the northeastern hill region of India were characterized using 28 RAPD and 30 ISSR markers (Verma et al., 2017). Thus, the molecular markers that have been developed represent a valuable resource to explore genetic linkage and gene localization.

### Tissue Culture

Tissue culture, a simple and powerful technique, is used to generate plants in artificial media under aseptic and controlled environments. Plant tissue culture has rapidly become an essential techniques for plant breeding, including rapid clonal propagation, haploid techniques, embryo culture, somatic embryogenesis, synthetic seed production, and callus culture (Kyte et al., 2013). Cruz-Martínez et al. (2017) successfully regenerated plants by inducing them from axenic nodal segments, leaves, and petioles of *S. edule* plants. Abdelnour-Esquivel and Engelmann (2002) developed a micropropagation protocol from the shoots of *S. edule*, and the results showed that using the shoots as explants can induce the production of high percentages of callus. Moreover, the hypocotyl was also used as explants to develop an efficient plant regeneration system in the tissue cultures of *S. edule*.

Depending on the different purposes and materials, the type of hormones in different tissue cultures is different. Axillary shoots were induced from axenic nodal segments in MS media supplemented with 0.1mg/l 6-benzylaminopurine (BA) (Katase, 1993; Masahiko, 1993). The leaf and petiole explants of *S. edule* were cultured on MS media supplemented with 0.1mg/l BA and 0.05mg/l gibberellic acid (GA3; Cruz-Martinez et al., 2017). Callus from hypocotyls was induced on MS media with 0.1mg/l indole acetic acid (IAA) and 1mg/l 6-BA. Adventitious bud formation from calli was obtained in MS media with 0.5mg/l IAA and 0.5mg/l 6-BA (Cruz-Martinez et al., 2017). GA3 plays an important role in promoting the elongation of regeneration buds (Amilineni et al., 2016). Moreover, different concentrations of auxin, IAA, and indole-3-butyric acid strongly induced the formation of roots from regenerated shoots.

Tissue culture of *S. edule* plant was first used to produce a considerable number of sterile seedlings to avoid using the fruit for sowing and reduce the cost. In addition, tissue culture is a powerful technique to study genetic transformations using *Agrobacterium tumefaciens*-mediated and particle bombardment methods. Although tissue culture systems of *S. edule* have been established, there are few reports on genetically modified *S. edule* plants.

## Nanotechnology

The application of nanotechnology in plants has the potential to alter conventional crop production systems, enabling the controlled release of agrochemicals, e.g., fertilizers, pesticides, and herbicides, and target-specific delivery of biomolecules, e.g., nucleotides, proteins, and activators (Ocsoy et al., 2018; Fiol et al., 2021). The *S. edule* plant has a valuable role in other sustainable methods for nanotechnology. Terrazas-Hernandez et al. (2015) revealed that the cellulose nanocrystals enhanced the properties of cast films made of the tuber starch in *S. edule* plants. Moreover, chayote extracts have been used to synthesize stable silver nanoparticles based on their antioxidant properties (Bag et al., 2018).

## Diseases and Pests

As an important vegetable crop in the world, one of the main limiting factors in marketing is the losses of yield and quality caused by fungal diseases that range from 15 to 25% (Alvarado et al., 1989; Narayanasamy, 2006). The goal of this review is to summarize the pests, diseases, and corresponding symptoms during the growth and development of *S. edule* (Table 2). The chayote is attacked by different pathogenic fungi, particularly *Ascochyta phaseolorum* and several species of *Fusarium*, *Macrophomina*, and *Colletotrichum*. Merlín et al. (2007) identified five different pathogen fungal blisters caused by *C. gloeosporioides*, anthracnose caused by *C. orbiculare*, reddish-purple mold caused by *F. oxysporum*, white mold caused by *Phytophthora capsici*, and acid rot caused by *Geotrichum* sp. Treatment with hot water and chlorine (1.5%) can effectively inhibit the occurrence and spread of diseases. Bezerra et al. (2016) reported that anthracnose on the fruits of *S. edule* plant was caused by *Colletotrichum brevisporum*, and further analysis found that the anthracnose occurred in all parts of the *S. edule* plant at any time during the growing season. Web blight, caused by *Thanatephorus cucumeris*, causes extensive leaf blight and webbing of the leaves, which were both associated with the presence of numerous microsclerotia on the dried leaves and petioles. Downy mildew disease caused by *Pseudoperonospora cubensis* on chayote was first reported in Taiwan (Ko et al., 2008). Chayote canker, caused by the bacterium *Xanthomonas campestris*, causes serious damage to the leaves of *S. edule*, which produces brown leaf spots surrounded by a yellow halo, and the central area of leaf spots often tear away during the late stage of canker disease (Dane et al., 1990). Chayote mosaic virus isolated from some accessions of *S. edule* in Costa Rica inhibits plant growth, produces deformed flowers, and decreases the amount of fruit set (Bernal et al., 2000). Alfalfa mosaic virus was found on chayote plants for the first time in Italy (Parrella et al., 2020).

**Table 2:** Diseases and pests in *S. edule*.

Biological diseases	Pathogen	Hazard site
**Fungal diseases**		
Leaf spot	Pseudomonas syringae pv. lachrymans	Leaf blade
Downy mildew	Trichoderma cubans	Leaf blade
Powdery mildew	Ascomycetes and Monocystis	Leaf blade, petiole, stem, fruit
Vine wilt	Microsporus of watermelon shell, partly known as Ascomycetes	Fruit, leaf, stem vine
Anthrax	Acanthopanax senticosus from the family A. semigeniculata	Leaf, stem, fruit
Black Star Disease	Trichosporidium parvum	Leaf blade
**Bacterial diseases**		
Ulcerative disease	The pathogen of the disease is the bacteria of the genus Rhizoma	Leaf blade
Viral diseases	CMV, CGMMV,WCMV, SLCV	Leaf, fruit
Mosaic virus	SQMV, MLCV, MVBMV, PMV, MWMV, WMV, TMV, BYMV	Leaf, fruit
Leaf spot	CCSV, CLSV, MNSV, WSMV, WLMV, ZYFV, TRV, PRSV	Leaf, fruit
**Pests**		
Whitefly	Homoptera whitefly	Leaf, fruit
Red Star	Leaf acarites of the genus Caryophyllaceae	Leaf blade
Thrips	Insect species tassel	Leaf, fruit
Aphids	General species	Leaf, fruit
**Non-biological diseases**		
Leaf fever	High temperature	Leaf blade

A potential threat for the *S. edule* crop is also pests, such as whiteflies and thrips (Johari and Lukman, 2017). They can damage the leaves and cause plant death in severe cases. Chemical, biological control, and strengthened field management can effectively reduce the occurrence of pests.

## Omics Research

Functional genomics is used to study how genes and intergenic regions of the genome contribute to different biological processes (Hieter and Boguski, 1997; Pevsner, 2015). With the advent of functional genomic research, the genome sequencing of the plants has been substantially accelerated, and a large amount of data has been generated (Morozova and Marra, 2008; Ondov et al., 2008). As an important squash vegetable, genomic, transcriptomic, and metabolomic analyses of the *S. edule* plant have been generated (Fu et al., 2021). A high-quality whole-genome sequence of *S. edule* with a size of 606.42Mb has been completed. The genome of *S. edule* is composed of 14 chromosomes and 28,237 predicted genes with 1,434 genes unique to *S. edule*. Moreover, 401.08Mb of repetitive sequences (65.94% of the genome) were identified using an analysis of the genome database, structural predictions, and the long terminal repeats (LTRs) that comprised the highest proportion of repetitive sequences. Transcriptomics and metabolomics are used to investigate the sequence information and metabolite changes in different tissues/organs or the same tissues/organs at the different developmental stages. In 2021, the transcriptome and metabolome of the fruits at different developmental stages of *S. edule* were established, and the results revealed that the genes involved in fruit texture, pigment, flavor, flavonoids, antioxidants, and plant hormones are expressed at different levels during chayote fruit development (Fu et al., 2021). To our knowledge, this transcriptome and metabolome are the first ones reported for the *S. edule* plant. Moreover, immature seeds also contain phytohormones (gibberellin and auxin), which are responsible for the rapid growth of seeds and fruits (Albone et al., 1984; Roy, 2000).

## Conclusions and Future Perspectives

*Saccharum edule* plant, as an important source of various nutrients and nutraceuticals, has the potential to be applied in the food, cosmetic, and pharmaceutical industries. With the increasing health consciousness, underutilized or neglected chayote crops have been studied more intensively owing to their rich nutraceuticals. However, many theoretical and technical problems persist in chayote research, including a lack of adequate germplasm resources, genetic breeding, and molecular biology. Future research should entail the collection of more germplasm resources of chayote and the establishment of efficient breeding platforms and systems. The whole-genome sequencing of chayote will contribute to an accurate understanding of the chayote genome. Re-sequencing can also accelerate the screening of germplasm resources, genetic evolution analyses, and the prediction of differential genes. Moreover, the study of expression patterns through chayote transcriptomes and proteomics should be conducted to reveal the functions of genes in chayote. With the development and application of molecular biology, research on active substances should focus on their synthesis and metabolic pathways, mechanisms of pathological regulation, and the popularization and application of research results.

## Author Contributions

X-JW, G-FT, Y-TP, L-HW, Y-RL, P-HM, and QL collected the manuscript data. X-JW, Y-TP, G-FT, QL, and L-HW wrote the manuscript. X-JW, G-FT, and P-HM approved the final manuscript. All authors have read and approved the final manuscript.

## Funding

This study was financially supported by Guizhou Science and Technology Support Project [Qiankehe Support (2019) 2257]; Guizhou High Level Innovative Talents Training, Hundred Level Talents Project [Qiankehe talent (2015) 4024]; Guizhou Science and Technology Plan Project [Qiankehe Support (2021) 210]; and Guizhou Science and Technology Plan Project [Qiankehe Support (2021) 211].

## Conflict of Interest

The authors declare that the research was conducted in the absence of any commercial or financial relationships that could be construed as a potential conflict of interest.

## Publisher’s Note

All claims expressed in this article are solely those of the authors and do not necessarily represent those of their affiliated organizations, or those of the publisher, the editors and the reviewers. Any product that may be evaluated in this article, or claim that may be made by its manufacturer, is not guaranteed or endorsed by the publisher.
